# Exploring aging interventions in human brain organoids

**DOI:** 10.18632/aging.203925

**Published:** 2022-02-28

**Authors:** Julio Aguado, Ernst J. Wolvetang

**Affiliations:** 1 Australian Institute for Bioengineering and Nanotechnology, The University of Queensland, Queensland 4072, Australia

**Keywords:** brain organoids, senolytics, senomorphics, brain aging, cellular senescence

Aging is a progressive physiological degeneration and represents the most important risk factor for the development of multiple human neurodegenerative disorders [1]. Therefore, determining the molecular drivers of aging is fundamental to understand brain aging comorbidities. Importantly, current evidence indicates that the progressive accumulation of senescent cells strongly contributes to brain degeneration and organismal aging [2].

In recent years, new strategies for targeting senescent cells have been developed to alleviate the systemic detrimental effects that arise from these cells. Some of these therapies are within the family of so-called senotherapeutics: drugs that either selectively eliminate senescent cells (senolytics) or that effectively suppress the senescence-associated secretory phenotype (SASP) responsible for driving chronic inflammation during aging (senomorphics). Until recently, their impact in the central nervous system had mostly been tested in mice where a number of studies have shown that senescent oligodendrocyte precursor cells, astrocytes and microglia are responsible for neurodegenerative phenotypes in mouse models of Alzheimer’s disease [3], tau-dependent pathology [4] and physiological murine aging [5], respectively. These studies used either the senolytics ABT-263 (a potent BCL inhibitor also known as Navitoclax) or a combination of Dasatinib and Quercetin (D+Q); a combination therapy where Q is a flavonol that inhibits the activity of mTOR and PI3K pathways, and D an anticancer drug and tyrosine kinase inhibitor. Importantly, the limitation of senotherapeutic drug assessment in the human brain strictly resides in the inability to examine the immediate and long-term action of these compounds *in situ* in human brain specimens.

Brain organoids (BOs) have become a valuable model platform for human brain disease modelling as they accurately model complex neuronal networks and to an extent recapitulate the cell diversity of the human brain [6]. This indeed is particularly relevant for the study of senescence in the brain, as biopsies from this tissue are exceptionally invasive and most reports in humans rely on *post mortem* evaluation.

Ataxia-Telangiectasia (A-T), a genome instability disorder caused by biallelic mutations in the ataxia-telangiectasia mutated (*ATM*) gene, is a premature aging syndrome that in many aspects recapitulates human brain aging [7]. In a recent paper we established a model of human accelerated brain aging using BOs generated from induced pluripotent stem cells derived from Ataxia-Telangiectasia (A-T) patient cells [8]. As compared to healthy wild type counterparts, A-T BOs display multiple hallmarks of aging, including SASP activation, lamin B1 downregulation, micronuclei accumulation, and increased senescence-associated β-galactosidase (SA-β-gal) activity; phenotypes that appeared enriched in astrocyte populations [8]. This observed premature aging phenotype was shown to be dependent on the cGAS-STING signalling axis, as inhibition of either cGAS or STING was sufficient to not only significantly reduce the SASP, but to also reduce the number of senescent cells as measured by CDKN1A and SA-β-gal positive cells [8]. Furthermore, this study indicates that targeting SASP using cGAS and STING inhibitors has impacts beyond inflammation and cellular senescence, as A-T BOs treated with this senomorphic interventions stimulate BO neuronal activity, prevent neuronal loss and overall help preserve organoid integrity [8].

The clinical significance of chronic inflammation and the impact of aging in driving disease progression - such as neurodegeneration - in the central nervous system of the aging population has to date remained largely unresolved. It is now acknowledged that improved understanding of the mechanisms that drive aging hallmarks can lead to the identification of novel therapeutic targets. This is an area where human BO models have the potential to exponentially expand the area of human brain aging. It is therefore conceivable that high throughput testing in BOs of known senotherapeutic and novel compounds may be used in the future to study mechanisms of aging as well as their capacity to alleviate multiple brain aging phenotypes.
